# Antimicrobial Stewardship Program Implementation, Perceptions, and Barriers in Zambia: A Cross-Sectional Study Among Healthcare Professionals

**DOI:** 10.3390/antibiotics14111094

**Published:** 2025-11-01

**Authors:** Steward Mudenda, Joseph Yamweka Chizimu, Victor Daka, Jimmy Hangoma, Kelvin Mwangilwa, Priscilla Gardner, Chikwanda Chileshe, Taona Sinyawa, Zoran Muhimba, Charles Chileshe, Sandra Diana Mwadetsa, Shikanga O-Tipo, Duncan Chanda, Maisa Kasanga, Geoffrey Mainda, Webrod Mufwambi, Samson Mukale, Andrew Bambala, Fusya Goma, Aubrey Chichonyi Kalungia, Yasuhiko Suzuki, Brian Godman, John Bwalya Muma, Roma Chilengi

**Affiliations:** 1 Zambia National Public Health Institute, Antimicrobial Resistance Coordinating Committee, Lusaka 10101, Zambia; mwangilwakelvin@yahoo.com (K.M.); priscillagardner82@gmail.com (P.G.); zmuhimba@yahoo.com (Z.M.); chichalesi2@gmail.com (C.C.); kasangaanita@gmail.com (M.K.); mukalesamson14@gmail.com (S.M.); chilengir@yahoo.com (R.C.); 2 Department of Pharmacy, School of Health Sciences, University of Zambia, Lusaka 10101, Zambia; webrod.mufwambi@unza.ac.zm (W.M.); ckalungia@unza.ac.zm (A.C.K.); 3 Public Health Department, School of Medicine, Copperbelt University, Ndola 10101, Zambia; dakavictorm@gmail.com; 4 Department of Pharmacy, School of Health Sciences, Levy Mwanawasa Medical University, Lusaka 10101, Zambia; jimmy.hangoma@lmmu.ac.zm; 5 Department of Biomedical Sciences, School of Veterinary Medicine, University of Zambia, Lusaka 10101, Zambia; chikchile@gmail.com; 6 Ministry of Fisheries and Livestock, Lusaka 10101, Zambia; taonasinyawa@gmail.com (T.S.); fusya.goma@mfl.gov.zm (F.G.); 7 Department of Disease Control, School of Veterinary Medicine, University of Zambia, Lusaka 10101, Zambia; jmuma@unza.zm; 8 Department of Infectious Diseases, University Teaching Hospital, Lusaka 10101, Zambia; duncanchanda@gmail.com (D.C.);; 9 Word Health Organization, Lusaka 10101, Zambia; mwadetsas@who.int (S.D.M.); otipos@who.int (S.O.-T.); 10 Food and Agriculture Organization, Lusaka 10101, Zambia; geoffrey.mainda@fao.org; 11 Division of Research Support, Institute for Vaccine Research and Development, Hokkaido University, Sapporo 001-0021, Japan; suzuki@czc.hokudai.ac.jp; 12 Department of Public Health Pharmacy and Management, School of Pharmacy, Sefako Makgatho Health Sciences University, Garankuwa, Pretoria 0208, South Africa; brian.godman@smu.ac.za; 13 Department of Pharmacoepidemiology, Strathclyde Institute of Pharmacy and Biomedical Sciences, University of Strathclyde, Glasgow G4 0RE, UK; 14 Antibiotic Policy Group, Institute for Infection and Immunity, City St. George’s, University of London, London SW17 0RE, UK

**Keywords:** antimicrobial stewardship, antimicrobial resistance, healthcare professionals, barriers, implementation, perceptions, Zambia

## Abstract

**Background/Objectives**: Antimicrobial stewardship programs (ASPs) play a vital role in combating antimicrobial resistance (AMR). However, their implementation in Zambia remains variable despite some notable progress. This study assessed healthcare professionals’ awareness of the Multisectoral National Action Plan (NAP) on AMR, alongside their perceptions, barriers, and implementation practices related to ASPs. **Methods**: A cross-sectional survey conducted between August and December 2024 included 364 healthcare professionals (HCPs) in 58 randomly selected public healthcare facilities in Zambia. Data were analysed using IBM SPSS 25.0. **Results**: Findings revealed that while 75.3% of respondents were aware of the Zambian NAP on AMS, only 68.1% of the respondents reported that their hospitals had established AMS committees. Conversely only 41.2% of the respondents stated that their hospitals possessed hospital-specific treatment guidelines. Encouragingly, 97.5% believed ASPs could enhance clinical outcomes and reduce AMR. Key barriers included limited funding (75.9%), inadequate IT infrastructure (64.1%), limited access to essential data (64%), and healthcare workforce shortages (53.8%). **Conclusions**: Whilst HCPs in Zambia demonstrated high awareness of the NAP and supported ASP implementation, systemic challenges hindered their consistent execution across health facilities. Gaps in treatment guideline development, AMR data usage, and the integration of antimicrobial susceptibility recording systems into clinical activities must be addressed to strengthen ASP efforts nationwide.

## 1. Introduction

Antimicrobial resistance (AMR) has emerged as one of the most pressing global public health challenges of the 21st century [1,2,3]. AMR compromises the effectiveness of antimicrobials, increasing morbidity and mortality as well as imposing significant economic strain on healthcare systems [1,4,5,6,7]. The persistent misuse and overuse of antimicrobials across the human, animal, and agriculture sectors continue to accelerate the emergence and spread of resistant pathogens [8,9,10,11,12,13], with AMR increasingly seen as the next pandemic unless multiple coordinated activities are undertaken [3,4,14,15,16]. This is particularly important in low- and middle-income countries (LMICs), including African countries, where AMR rates are high and growing [1,15,17]. Zambia is no exception, with currently high rates of AMR, including for commonly used antibiotics [10,18,19].

In response to this escalating threat, several global, regional, and national initiatives have been launched. The World Health Organization (WHO) introduced the Global Action Plan on AMR in 2015, urging member states to develop their national action plans (NAPs) and implement sustainable interventions to improve antimicrobial use, including antimicrobial stewardship programs (ASPs) [20,21]. However, concerns have been raised regarding the implementation of NAPs in LMICs, including those in Africa, due to personnel and resource issues, as well as underdeveloped healthcare infrastructures, which include a lack of laboratory capacity for routine analysis of local and regional resistance patterns [21,22,23,24,25,26,27].

ASPs refer to coordinated interventions designed to promote the appropriate use of antimicrobials, improve patient outcomes, reduce AMR, and decrease the spread of infections caused by multidrug-resistant organisms [28,29,30,31,32,33]. For ASPs to be effective, a collaborative, multidisciplinary approach is essential, engaging a range of professionals, including physicians, pharmacists, nurses, biomedical scientists, and infection prevention specialists [34,35,36]. The success of ASPs is closely underpinned by their integration into institutional structures, reinforced through comprehensive policies, robust standardised treatment guidelines, surveillance and diagnostic systems, and continuous professional capacity-building initiatives [37,38,39]. Recently, there have been concerns to promote the WHO’s Access, Watch, and Reserve (AWaRe) framework, which guides rational antibiotic use, with targets now requiring at least 70% of prescriptions to come from Access antibiotics [40,41,42,43]. Despite the 2022 AWaRe guidance book and growing integration of AWaRe-based quality targets into ASPs, excessive use of Watch and Reserve antibiotics continues to drive AMR globally, contributing to an estimated 659,000 child deaths in Africa [41,44,45,46,47]. This challenge is evident in many LMICs, including Zambia, where high use of Watch group antibiotics persists despite some facilities showing better prescribing practices [46,48,49,50,51,52,53,54].

Implementing AMS programs in LMICs, including Zambia, has been a challenge due to systemic limitations, including underdeveloped healthcare infrastructure, constrained financial resources, limited laboratory capacity, and shortages of key trained personnel [10,17,22,55]. There have also been concerns with limited knowledge and activities regarding antibiotics, AMR and ASPs among prescribers across the sectors in LMICs, including Zambia [56,57]. However, this is changing with multiple ASPs now being undertaken across Africa [22,33,58,59]. Zambia launched its ten-year NAP on AMR (2017–2027) in 2017, setting forth strategic priorities that include strengthening knowledge through surveillance and research, optimising antimicrobial use, and improving awareness and understanding of AMR across sectors [60].

Central to the success of ASPs are the commitment and knowledge of healthcare professionals (HCPs) [61,62,63], whose knowledge, attitudes, and practices significantly influence antimicrobial prescribing patterns, infection control strategies, and the implementation of AMS interventions [64,65]. Therefore, evaluating HCPs’ awareness of AMS principles, understanding the barriers they encounter in implementing ASPs, and identifying institutional support gaps are critical steps towards designing context-specific interventions to improve the future use of antibiotics in Zambia. We are aware that some studies have been undertaken in Zambia to assess current knowledge and attitudes towards antimicrobial use (AMU), AMR and ASPs [64,66]. However, given the previous concerns of gaps in ASP awareness and implementation in some studies in Zambia and the ongoing goals of the Zambian NAP to reduce AMR, we believed it was imperative to increase our understanding of institutional preparedness for ASP implementation in Zambia, including revealing any vital evidence gaps that impede effective policy translation. Given this, this study assessed the level of awareness of the Zambian NAP on AMR, perceptions, and barriers to ASP implementation among HCPs in Zambia. By assessing HCPs’ perspectives and current institutional practices, the study sought to generate insights that inform capacity-building initiatives, support policy implementation, and reinforce antimicrobial stewardship (AMS) efforts in Zambia.

## 2. Results

A total of 364 HCPs participated in the study. The majority were clinicians (27.5%), nursing professionals (26.6%), and pharmacy professionals (22.0%) (Table 1). Most respondents were aged 25–34 years (54.4%), with female respondents slightly outnumbering males (51.9%). In terms of professional experience, 66.5% had over five years of experience in their current specialty, indicating a predominantly experienced and mid-career workforce (Table 1).

Most of the respondents (75.3%) were aware of the Zambian NAP on AMR, providing a strong foundation for its effectiveness. The study also found that 68.1% of the respondents reported that their hospitals had an AMS committee, and only 66.2% stated that their hospitals had a policy for documenting antibiotic prescriptions, indicating areas for improvement. Additionally, only 41.2% of the respondents stated that their hospitals had hospital-specific treatment guidelines (Table 2). Notably, a considerable proportion of respondents were uncertain about the existence of these ASP components, suggesting potential gaps in communication or documentation.

Table 3 summarises the responses of HCPs (N = 364) to statements regarding the perceived importance of AMS activities. Regarding whether AMS activities would improve patients’ clinical outcomes, the vast majority of respondents (97.5%) either strongly agreed (80.2%) or agreed (17.3%) with this statement. This highlights a high level of awareness among HCPs regarding the clinical benefits of ASPs. Similarly, 97.6% of respondents agreed or strongly agreed that ASPs are a vital strategy to combat AMR. A combined 97% agreement rate indicates that most HCPs believed implementing ASPs could reduce unnecessary healthcare expenditure by minimising ineffective treatments and resistance-related complications.

Overall, the respondents significantly agreed (*p* < 0.001) with all the perception statements, indicating they viewed AMS activities as positively impacting the various aspects of AMR.

This study found that the main barriers to implementation of ASPs in Zambian hospitals included a lack of funding, inadequate IT support, limited resources for data collection, shortages of HCPs, and a lack of awareness of ASP by hospital administration (Table 4).

## 3. Discussion

This study aimed to assess the awareness of the Zambia NAP on AMR, perceptions, barriers, and implementation of ASP initiatives among HCPs in hospitals in Zambia. Overall, just over two-thirds of hospitals had an AMS committee, even though 75.3% of the respondents were familiar with Zambia’s NAP on AMR. Furthermore, the study found that 68.1% of the respondents reported that their facilities had AMS committees, and only 34.3% of respondents stated that their hospitals utilised AMR monitoring data to guide antibiotic choices. This is a concern with the lack of AMR surveillance limiting public health action and interventions. However, the findings in Zambia are similar to other LMICs, including other African countries, where comprehensive population-based surveillance of AMR, alongside data entry, was lacking and largely fragmented [67,68]. In addition to the observed challenges, we found a lack of software for recording antimicrobial susceptibility results as reported by 28.6% of the respondents, underscoring significant gaps in AMS infrastructure. These findings suggest that while awareness exists, translation into consistent practice and policy adherence remains a challenge. Previous findings in Zambia have also reported inadequate laboratory capacity to conduct microbiological tests and surveillance of AMR [69].

Despite these challenges, nearly all respondents agreed that ASPs would improve clinical outcomes and help reduce AMR. However, the main identified barriers to ASP implementation included limited funding, reported by approximately three-quarters of respondents; inadequate IT infrastructure, noted by nearly two-thirds; and restricted access to critical data, cited by a similar proportion. These findings suggest that while awareness exists, translation into consistent practice and policy adherence remains a challenge. Previous studies conducted in Zambia have also reported inadequate laboratory capacity to conduct microbiological tests and surveillance of AMR [69]. Insufficient funding continues to be a major barrier to the implementation of the ASPs [70]. A systematic analysis of AMR in the WHO African region noted that population-based surveillance is often absent, with data scarce due to inadequate laboratory infrastructure and fragmented reporting systems [17]. These findings underscore systemic gaps in laboratory infrastructure, digital health capacity, and human resources, which must be addressed across Africa, including Zambia, going forward to enable real-time AMR monitoring to inform antimicrobial policy and address rising AMR rates.

Additionally, just over half of the respondents pointed to a shortage of HCPs as a significant constraint to implementing ASPs, which is similar to other countries [39,55,71,72,73]. Alongside this, the lack of dedicated multidisciplinary ASP teams is among the main barriers to implementation of ASPs in hospitals [71,72,74]. Having said this, the presence of AMS committees and education programs in many health facilities as reported by the majority of HCPs indicates that foundational ASP components were in place in hospitals in Zambia at the time of the survey, similar to previous evidence [64]. This is critical in ensuring the establishment of functional ASPs in hospitals [35,75], with functional AMS committees and ASPs key to promoting the rational use of antibiotics in hospitals to meet UN GA targets [28,30,39,43,61,76,77].

Whilst encouraging, the overall awareness of the NAP on AMR was high among participating HCPs in our study, critical gaps remain in the operationalisation of ASPs among health facilities in Zambia, as reported earlier [78]. This pattern reflects similar challenges reported in other LMICs, where ASP implementation often lags behind policy directives due to systemic and infrastructural limitations of the NAP on AMR [62,72,78,79,80,81,82,83]. The disconnection between policy and practice could reflect a gap in the interpretation of the NAP on AMR by HCPs in previous studies [23,84].

In this study, another identified concern was the limited availability of hospital-specific STGs. This was built on a similar study in Zambia conducted among district hospitals, which found that while STGs were present in some facilities, overall compliance with the STGs ranged from 0% to 57% [53]. In addition, a study conducted in the African Union found that only 20 of the 55 countries in the Union had STGs [85]. The limited availability and poor use of STGs have far-reaching public health implications. The recent availability of the WHO AWaRe guidance covering the treatment of 35 infectious diseases across all sectors provides a robust platform going forward to address concerns with the lack of STGs [41,45,86]. Pertinent guidelines can subsequently be amended based on local resistance patterns. The next stages involved their active dissemination and monitoring. Concurrent with this, hospital HCPs must be trained not only on AMS principles but also on the practical application of these guidelines in clinical settings [65].

Encouragingly, HCPs in this study strongly believed in the effectiveness of ASPs in improving clinical outcomes (97.5%), reducing resistance (97.6%), and enhancing cost-effectiveness (97%), which was an improvement over previous studies in Zambia [73]. These high perception scores are consistent with findings from similar surveys in Kenya, Uganda and Nigeria, where HCPs recognised the value of AMS; however, they cited operational and contextual challenges as barriers to full implementation [87,88,89]. In addition, in this study, only a relatively small proportion of respondents viewed a lack of awareness among prescribers or opposition from them as barriers to ASPs, suggesting that the healthcare workforce in Zambia is receptive to AMS principles. These barriers have been reported in previous studies and imped progress made in addressing AMR [73,90,91]. This presents an opportunity to leverage the existing support to ASPs among HCPs through targeted investments in infrastructure, leadership, and accountability mechanisms.

In this study, some key barriers to implementing ASPs included inadequate funding, lack of IT support, limited access to data, shortage of HCPs, and lack of awareness of ASPs by administration staff. These barriers reflect well-documented constraints in LMICs, where AMS implementation is often hampered by weak laboratory capacity, inadequate leadership support, lack of electronic health records, and limited financial and human resources [22,57,62,81,92,93,94]. The absence of essential AMS enablers such as reliable microbiological diagnostics and routine antimicrobial use audits compromises the ability to monitor resistance patterns and optimise prescribing practices [71,95]. This further complicates the management of patients who may need timely interventions. Similarly, a study conducted in Saudi Arabia found that the major barriers to implementation of ASPs were a lack of internal policy/guidelines and specialised AMS information resources, inadequate administrative awareness of ASPs, lack of AMS personnel, limited training opportunities, time limitation, lack of confidence, and limited funding [73]. Consequently, there is a need to strengthen AMS interventions and other strategies to combat AMR in Zambia and across LMICs [33,38,75,96,97,98,99].

We are aware that this study has some limitations. First, the use of convenience sampling limits the generalizability of the findings to all HCPs in Zambia. Although facilities were randomly selected, recruitment of participants within facilities was based on convenience sampling, which may introduce selection bias and affect representativeness across professional groups. Second, the cross-sectional design captures responses at a single point in time and may not reflect ongoing changes in AMS awareness and implementation. Third, the study relied on self-reported data, which is subject to social desirability and recall bias. Moreover, the study did not assess actual AMS practices or antimicrobial prescribing patterns, which would provide a more comprehensive understanding of stewardship performance. Fourth, while the survey covered multiple cadres and facilities across Zambia, the inclusion of only public healthcare facilities limits the generalizability of findings to private sector and community-based settings. Fifth, the study did not directly measure antibiotic prescribing practices or link responses to microbiological or pharmacy data, which restricts the ability to validate reported stewardship activities. Sixth, variations in ASP implementation between rural and urban healthcare settings were not accounted for during the analysis. Lastly, there was an uneven distribution of respondents across cadres (clinicians, nurses, pharmacists, biomedical scientists), as well as possible over-representation of urban facilities compared to rural ones, limiting generalizability.

Despite these limitations, we believe the study provides valuable insights into ASP implementations in health facilities in Zambia. Inclusion of a wide range of HCPs, including clinicians, nurses, pharmacists, biomedical scientists, public health officers, and allied health workers, offered diverse perspectives on AMS implementation. Additionally, addressing AMR and AMS aligns with national and global health priorities, particularly the WHO GAP and Zambia’s NAP on AMR. Further, the study provides current, context-specific evidence on the awareness, implementation status, and barriers of AMS in Zambia, filling a critical knowledge gap. Furthermore, the research highlights systemic and operational barriers such as a lack of funding, inadequate IT infrastructure, and data limitations, which are essential for designing targeted interventions. A sample of 364 HCPs strengthens the reliability and generalizability of the findings within the Zambian context. The study also revealed strong healthcare professional support for AMS, providing a solid foundation for advocacy, training, and policy development. The findings directly inform Zambia’s AMR policy landscape by identifying implementation gaps and opportunities for strengthening institutional ASP capacity. Finally, the study provided valuable baseline data that can be used to monitor trends in ASP implementation and effectiveness over time.

The policy implications and recommendations of this study are provided in Table 5. This study shows that while awareness of ASPs in Zambia is high among HCPs, implementation is limited. Policymakers should strengthen enforcement of the Zambia NAP on AMR, increase funding, improve IT and laboratory capacity, and align antibiotic use with WHO AWaRe targets. At the practice level, hospitals should develop and adapt treatment guidelines, promote inter-professional collaboration, and conduct regular audits to ensure rational antibiotic prescribing. Addressing workforce shortages and leveraging HCPs’ positive perceptions of ASPs will be key to strengthening stewardship and reducing inappropriate antibiotic use.

## 4. Materials and Methods

### 4.1. Study Design and Setting

This study employed a cross-sectional design conducted between August 2024 and December 2024 in Zambia. The survey targeted HCPs in facilities that included primary, secondary and tertiary care settings. Only HCPs registered with the Health Professions Council of Zambia and the Nursing Council of Zambia were eligible to participate in the study. The study aimed to assess HCPs’ awareness of the Zambian NAP on AMR, perceptions and barriers to the implementation of ASPs in Zambia.

### 4.2. Study Population and Sampling

The study population comprised registered HCPs in Zambia, including medical doctors, clinical officers, nurses, pharmacy personnel (pharmacists & pharmacy technologists), biomedical scientists, public health officers, and other allied health professionals. A stratified sampling approach was subsequently employed. Firstly, a list of all public health facilities in the country was obtained from the Ministry of Health to serve as the sampling frame. Of these facilities, 100 were selected as sites to establish and implement ASPs across the country. Hence, a random selection of the facilities was made, ensuring regional representation. Within each selected facility, convenient sampling was undertaken to select respondents across different healthcare professions, including medical doctors, clinical officers, nurses, pharmacy personnel, biomedical scientists, public health officers, and those in administration. The respondents were recruited conveniently based on their professional role and availability during the survey period. From a total of 100 facilities where the Zambia Antimicrobial Resistance Coordinating Committee (AMRCC) planned to instigate ASPs, a total of 58 (46 secondary and 12 tertiary hospitals) hospitals were randomly selected to reduce bias. The stratified facilities included secondary and tertiary hospitals, which were selected for the initiation of ASPs. The random approach ensured representation from diverse facilities and geographic regions.

### 4.3. Inclusion and Exclusion Criteria

Eligible respondents were those directly or indirectly involved in antimicrobial prescribing, dispensing, administration, or monitoring and who provided informed consent to participate. Respondents were excluded if they did not provide informed consent or withdrew from the study before completion.

### 4.4. Data Collection Tool and Procedure

Data were collected using a structured, self-administered questionnaire developed based on a similar previous study among HCPs [100]. The questionnaire was designed in English, the official language used in healthcare settings in Zambia. It consisted of five sections, including demographics (age, gender, profession and years of experience), awareness of the NAP on AMR and ASPs, implementation of ASP components, perceptions of the importance and effectiveness of ASPs and the perceived barriers to ASP implementation (See Supplementary Materials).

The data collection tool was validated to ensure clarity, consistency, and face validity, with input from public health experts from the Zambia AMRCC and academia before the study. This was followed by a pilot study using a sample of 20 HCPs. Feedback from the pilot study was used to refine the questionnaire before full-scale deployment, but did not form the final analysis of the study.

Questionnaires were administered in person by trained research assistants at each public health facility, ensuring standardised delivery and data collection procedures. Respondents completed the questionnaires anonymously and returned them in sealed envelopes to maintain confidentiality.

### 4.5. Data Management and Statistical Analysis

The completed questionnaires were checked for completeness and consistency before data entry into Microsoft Excel version 2013 and then exported to IBM SPSS Statistics (Version 25.0; IBM Corp., Armonk, NY, USA) for analysis. Descriptive statistics were used to summarise respondents’ demographic characteristics and the distribution of responses across awareness, implementation, perception, and barrier domains. Differences in responses were assessed with the use of the chi-square. Statistical significance was set at *p* < 0.05, and findings were presented using tables and charts.

## 5. Conclusions

This study highlights that although HCPs in Zambia demonstrated strong awareness of the NAP on AMR and ASP activities, and appreciate their role in combating AMR, notable deficiencies persist in the practical implementation of ASPs within hospitals in Zambia. Key structural barriers, including insufficient funding, limited IT infrastructure, and lack of access to surveillance data, significantly compromise the effectiveness of AMS strategies in Zambia. Targeted investment in digital health programs, reinforcing policy frameworks, and delivery of sustained capacity-building and education initiatives will be key to bridging these gaps in Zambia and similar LMIC settings.

## Figures and Tables

**Table 1:** Healthcare Professionals Demographic Characteristics (N = 364).

Category	Subcategory	Frequency	Percent
Distribution of HCPs	Clinicians	100	27.5
	Pharmacy Professionals	80	22.0
	Nursing Professionals	97	26.6
	Biomedical Science Professionals	49	13.5
	Public & Environmental Health	20	5.5
	Microbiologists	6	1.6
	Health Information/Administration Staff	5	1.4
	Other Allied Health Professionals	7	1.9
	Total	364	100.0
Age (Years)	18–24	3	0.8
	25–34	198	54.4
	35–44	99	27.2
	45–54	56	15.4
	55 and above	8	2.2
	Total	364	100.0
Gender	Male	175	48.1
	Female	189	51.9
	Total	364	100.0
Experience in Current Specialty	Less than 1 year	15	4.1
	1–5 years	107	29.4
	Above 5 years	242	66.5
	Total	364	100.0
Hospital Classification	Secondary	46	79.3
	Tertiary	12	20.7
	Total	58	100
HCPs by Hospital	Secondary	245	67.3
	Tertiary	119	32.7
	Total	364	100

**Table 2:** Implementation of Antimicrobial Stewardship Program Components in Zambian Hospitals (N = 364).

ASP Component	Yes n (%)	No n (%)	Not Sure n (%)
Awareness of National Action Plan (2017–2027)	274 (75.3)	90 (24.7)	0.0
The hospital has an AMS Committee	248 (68.1)	74 (20.3)	42 (11.6)
Policy on documenting dose, duration, and indication for antibiotics	241 (66.2)	63 (17.3)	60 (16.5)
Hospital-specific treatment guidelines based on national/local susceptibility	150 (41.2)	134 (36.8)	80 (22.0)
Use of AMR surveillance reports	125 (34.3)	112 (30.8)	127 (34.9)
Software to record antimicrobial susceptibility results	104 (28.6)	137 (37.6)	123 (33.8)
Availability of antimicrobial use reports	139 (38.2)	103 (28.3)	122 (33.5)
Access to evidence-based medicine during care	212 (58.2)	73 (20.1)	79 (21.7)
Education for prescribers on optimal prescribing and resistance	241 (66.2)	71 (19.5)	52 (14.3)

**Table 3:** Perceptions of Healthcare Professionals on AMS (N = 364).

Perception Statement	Response Option	Frequency	%
AMS will improve patients’ clinical outcomes	Strongly agree	292	80.2
	Agree	63	17.3
	Neutral	8	2.2
	Strongly disagree	1	0.3
AMS will reduce antimicrobial resistance	Strongly agree	294	80.8
	Agree	61	16.8
	Neutral	8	2.2
	Disagree	1	0.3
AMS improves the cost-effectiveness of healthcare	Strongly agree	267	73.4
	Agree	86	23.6
	Neutral	10	2.7
	Disagree	1	0.3
AMS improves collaboration among healthcare providers	Strongly agree	245	67.3
	Agree	100	27.5
	Neutral	19	5.2

**Table 4:** Reported Barriers to AMS Program Implementation in Zambian Hospitals (N = 364).

Barrier	Response	Frequency	(%)
Lack of sufficient healthcare providers	Strongly agree	75	20.6%
	Agree	121	33.2%
	Neutral	78	21.4%
	Disagree	69	19.0%
	Strongly disagree	21	5.8%
Lack of funding	Strongly agree	140	38.5%
	Agree	136	37.4%
	Neutral	54	14.8%
	Disagree	31	8.5%
	Strongly disagree	3	0.8%
Hospital administration is unaware of AMS programs	Strongly agree	37	10.2%
	Agree	46	12.6%
	Neutral	63	17.3%
	Disagree	137	37.6%
	Strongly disagree	81	22.3%
Prescribers are unaware of AMS programs.	Strongly agree	44	12.1%
	Agree	77	21.2%
	Neutral	73	20.1%
	Disagree	120	33.0%
	Strongly disagree	50	13.7%
Opposition from prescribers	Strongly agree	36	9.9%
	Agree	85	23.4%
	Neutral	117	32.1%
	Disagree	100	27.5%
	Strongly disagree	26	7.1%
Lack of IT support	Strongly agree	65	17.9%
	Agree	168	46.2%
	Neutral	71	19.5%
	Disagree	47	12.9%
	Strongly disagree	13	3.6%
Lack of resources to get the needed data	Strongly agree	84	23.1%
	Agree	149	40.9%
	Neutral	71	19.5%
	Disagree	45	12.4%
	Strongly disagree	15	4.1%

**Table 5:** Policy Implications and Recommendations Based on the Study Findings.

Key Finding	Policy Implications	Practice Recommendations
High awareness of ASPs among HCPs, but poor implementation	Incorporate AMS into national health priorities and policies; strengthen enforcement of the Zambia National Action Plan (NAP) on AMR	Enhance training and continuous professional development of HCPs on ASP implementation
Limited hospital-specific treatment guidelines and surveillance data use	Develop, update, and enforce standardised treatment guidelines (STGs) informed by local AMR data	Support hospital-level AMS committees to adapt STGs for local use
Major barriers: lack of funding, IT support, and resources for data	Increase dedicated government and donor funding for AMS activities and infrastructure	Invest in laboratory and IT systems for AMR data collection and reporting
Workforce shortages	Advocate for recruitment and retention policies to expand health workforce capacity.	Train multidisciplinary teams to implement AMS at the facility level
Positive perceptions of ASP benefits among HCPs	Leverage HCPs’ support to integrate AMS into routine practice	Promote inter-professional collaboration and mentorship in AMS activities
High use of Watch antibiotics and gaps in adherence to AWaRe targets	Align procurement and prescribing policies with WHO AWaRe targets (≥70% Access group use)	Conduct regular audits and feedback to ensure rational antibiotic prescribing

## Data Availability

The data supporting the reported results can be made available on reasonable request from the corresponding author.

## Supplementary Materials

The following supporting information can be downloaded at: https://www.mdpi.com/article/10.3390/antibiotics14111094/s1, Questionnaire.

## Abbreviations

The following abbreviations were used in this manuscript:

AMR	Antimicrobial Resistance
AMS	Antimicrobial Stewardship
GRADE	Grading of Recommendations, Assessment, Development and Evaluation
HCP	Healthcare Professionals
IBM	International Business Machine
IT	Information Technology
KAP	Knowledge, Attitudes, and Practices
LMICs	Low and Middle-Income Countries
NAP	National Action Plan
NY	New York
SPSS	Statistical Package for Social Sciences
STGs	Standard Treatment Guidelines
TDRC	Tropical Diseases Research Centre
WHO	World Health Organisation
USA	United States of America
