# Supplementary material for: Antimicrobial Stewardship Program Implementation, Perceptions, and Barriers in Zambia: A Cross-Sectional Study Among Healthcare Professionals
Source: Antibiotics (Basel). 2025 Nov 1;14(11):1094. doi: 10.3390/antibiotics14111094 (PMC12649657; doi:10.3390/antibiotics14111094)
Supplement: Supplementary file 1 [file antibiotics-14-01094-s001.zip › antibiotics-3806321-supplementary.pdf]

**Antimicrobial Stewardship Program Implementation, Perceptions, and Barriers in Zambia: A Cross-Sectional Study among Healthcare Professionals**

Dear participant

This study is assessing healthcare professionals' awareness of the Multisectoral National Action Plan (NAP) on AMR, alongside their perceptions, barriers, and implementation practices related to Antimicrobial Stewardship Programs.

For confidentiality, the surveys will not include information identifying your personal identity and will be used only for research purposes.

**Part 1: Demographic characteristics of healthcare providers**

1. Age: ________________ (years)
2. Gender:
   a. Male
   b. Female
3. **Healthcare discipline**
   a. Clinicians
   b. Pharmacy Professionals
   c. Nursing Professionals
   d. Biomedical Science Professionals
   e. Public & Environmental Health
   f. Microbiology Professionals
   g. Health Information/Administration Staff
   h. Other Allied Health Professionals
4. **Experience in your current specialty or profession:**
   a. Less than 1 year
   b. 1-5 years
   c. above 5 years
5. Name of the hospital: ______________________

6. **Are you aware about the presence of the National Action Plan on antimicrobial stewardship (2017–2027) in Zambia?**
    a. Yes
    b. No

**Part 2:** Assessment of antimicrobial stewardship practice in hospital settings

Organizational structure

7. **Does your hospital have an antimicrobial stewardship committee?**
    a. Yes
    b. No
    c. Not sure
8. **Does your hospital have a policy that requires prescribers to document in the medical record of during entry a dose, duration, and indication for all antibiotic prescriptions?**
    a. Yes
    b. No
    c. Not sure
9. **Based on national guidelines and local susceptibility, does your hospital have a hospital-specific treatment recommendation (guideline)?**
    a. Yes
    b. No
    c. Not sure
10. **Please choose the available antibiotic stewardship strategy in your hospitals**

(Multiple answers is possible)

a. Treatment guidelines
b. Surgical prophylaxis guidelines
c. Antimicrobial cycling
d. Prospective audit feedback
e. Antimicrobial formulary
f. Intravenous-to-oral switch guidance
g. Empiric usage form
h. Restricted antimicrobial list
i. Streamlining or de-escalation of therapy
j. Pre-authorized pharmacy-driven dose optimization (e.g., automatic renal dose adjustments, intravenous-to-oral conversions, etc.)

k. Infectious diseases/microbiology advice by telephone
l. Infectious diseases/microbiology advice on ward rounds
m. Separate antimicrobial chart or section

**11. Does your hospital use antimicrobial resistance levels/surveillance reports?**

a. Yes
b. No
c. Not sure

**12. Does your facility have software to record antimicrobial susceptibility results?**

a. Yes
b. No
c. Not sure

**13. Does your facility have any antimicrobial use reports?**

a. Yes
b. No
c. Not sure

**14. Does your hospital provide access to literature or evidence-based medicine for medical staff while delivering care?**

a. Yes
b. No
c. Not sure

**15. Does your stewardship program provide education to prescribers and other relevant staff on optimal prescribing, adverse reactions from antibiotics, and antibiotic resistance?**

a. Yes
b. No
c. Not sure

**Part 3:** The following questions are about perceptions on AMS. Perceived importance of antimicrobial stewardship.

**16. Antimicrobial stewardship will improve patient's clinical outcomes**

a. Strongly agree
b. Agree
c. Neutral
d. Disagree
e. Strongly disagree

**17. Antimicrobial stewardship will reduce antimicrobial resistance**

a. Strongly agree

b. Agree
c. Neutral
d. Disagree
e. Strongly disagree

**18. Antimicrobial stewardship improves the cost-effectiveness of health care sectors**

a. Strongly agree
b. Agree
c. Neutral
d. Disagree
e. Strongly disagree

**19. Antimicrobial stewardship improves the collaboration between healthcare providers**

a. Strongly agree
b. Agree
c. Neutral
d. Disagree
e. Strongly disagree

**Part 4:** The following concern the barriers of implementing AMS programs at your facility. Barriers to delivering a functional and effective antimicrobial stewardship Please choose the level of agreement with the following statements, reflecting the barriers of antimicrobial stewardship implementation in the hospital setting.

**20. Lack of sufficient healthcare providers**

a. Strongly agree
b. Agree
c. Neutral
d. Disagree
e. Strongly disagree

**21. Lack of funding**

a. Strongly agree
b. Agree
c. Neutral
d. Disagree
e. Strongly disagree

**22. The hospital administration is not aware of AMS program**

a. Strongly agree
b. Agree

c. Neutral
d. Disagree
e. Strongly disagree

**23. The antimicrobial Prescribers are not aware of AMS program**

a. Strongly agree
b. Agree
c. Neutral
d. Disagree
e. Strongly disagree

**24. Opposition from prescribers**

a. Strongly agree
b. Agree
c. Neutral
d. Disagree
e. Strongly disagree

**25. Lack of information technology support**

a. Strongly agree
b. Agree
c. Neutral
d. Disagree
e. Strongly disagree

**26. Lack of resources to get the needed data**

a. Strongly agree
b. Agree
c. Neutral
d. Disagree
e. Strongly disagree
